# Moderate Neonatal Stress Decreases Within-Group Variation in Behavioral, Immune and HPA Responses in Adult Mice

**DOI:** 10.1371/journal.pone.0001015

**Published:** 2007-10-10

**Authors:** Simone Macrì, Paolo Pasquali, Luca Tommaso Bonsignore, Stefano Pieretti, Francesca Cirulli, Flavia Chiarotti, Giovanni Laviola

**Affiliations:** 1 Section of Behavioural Neuroscience, Department of Cell Biology and Neuroscience, Istituto Superiore di Sanità, Roma, Italy; 2 Department of Food Safety and Veterinary Public Health, Istituto Superiore di Sanità, Roma, Italy; 3 Department of Drug Research and Evaluation, Istituto Superiore di Sanità, Roma, Italy; James Cook University, Australia

## Abstract

**Background:**

The significance of behavioral neuroscience and the validity of its animal models of human pathology largely depend on the possibility to replicate a given finding across different laboratories. Under the present test and housing conditions, this axiom fails to resist the challenge of experimental validation. When several mouse strains are tested on highly standardized behavioral test batteries in different laboratories, significant strain×lab interactions are often detected. This limitation, predominantly due to elevated within-group variability observed in control subjects, increases the number of animals needed to address fine experimental questions. Laboratory rodents display abnormal stress and fear reactions to experimental testing, which might depend on the discrepancy between the stability of the neonatal environment and the challenging nature of the adult test and housing conditions.

**Methodology/Principal Findings:**

Stimulating neonatal environments (e.g. brief maternal separations, increased foraging demands or maternal corticosterone supplementation) reduce stress and fear responses in adulthood. Here we tested whether reduced fearfulness associated with experimental testing would also reduce inter-individual variation. In line with our predictions, we show that a moderate elevation in neonatal corticosterone through maternal milk significantly reduces fear responses and inter-individual variability (average 44%) in adult mouse offspring.

**Conclusions/Significance:**

We observed reduced variation in pain perception, novelty preference, hormonal stress response and resistance to pathogen infection. This suggests that the results of this study may apply to a relatively broad spectrum of neuro-behavioral domains. Present findings encourage a reconsideration of the basic principles of neonatal housing systems to improve the validity of experimental models and reduce the number of animals used.

## Introduction

An accepted principle in science is that the outcomes of a given study should be reproducible in independent facilities if experimental conditions were kept constant. In a meticulously controlled study, Crabbe and colleagues [1] tested this assumption by evaluating eight different mouse strains on a highly standardized behavioral test battery in three independent facilities. Although strong genetic differences were generally preserved across labs, a considerable number of effects were dependent on the specific facility in which the test was conducted. For example, compared to wild types in the elevated plus-maze test, 5-HT_1B_ knockout mice had greater activity (measured as the number of entries in the closed arms) in Portland, tended to have less activity in Albany, and did not differ in Edmonton. In a partial replication of the study, Wolfer and colleagues [2] tested whether, compared to animal facility reared controls, mice reared in environmentally enriched cages would show higher [3] or lower between-lab experimental variability. Wolfer and colleagues [2] observed that mice reared under standard conditions and mice reared in enriched conditions showed an indistinguishable degree of between-lab consistency. Within-group variability was able to explain about 60% of experimental variance, thus indicating that inter-individual variation is the main source of experimental noise in behavioral studies [2].

It has been proposed that the elevated variability observed in captivity [4] stems from maladaptive adjustments to the laboratory environment shown by rodents [5]. The latter may depend on a poor predictive value of the neonatal life conditions as to the characteristics of the adult environment [6]. Many organisms, including plants and mammals, adjust their response systems according to the characteristics of the neonatal environment [7]. In turn, phenotypic success (expressed in terms of Darwinian *fitness* [8]) depends on the predictive value of the early *milieu* as to the characteristics of the adult habitat. Laboratory animals develop within the same constraints. Therefore, how well adapted an animal will be to the adult environment depends on how well the neonatal features reflect the adult laboratory ‘habitat’. Similar to natural conditions, newborn laboratory rats and mice spend their first–highly plastic–weeks of life under the very stable and safe conditions of the nest. However, in natural conditions maternal nest attendance depends on the environmental challenges encountered by the dams [9] (e.g. predator pressure, foraging and climate conditions, social competition). According to the maternal mediation hypothesis [10], patterns of maternal care, nest attendance and mother-offspring hormonal transfer will inform pups as to the characteristics of their future environment [5], [11], [12]. Developing offspring will in turn regulate their neurobehavioral systems according to this mode of information transfer [5], [11]. Thus, the more closely the patterns of maternal care predict the challenges of the adult environment, the better the offspring will be adapted to adult life conditions. Laboratory mouse dams are in constant contact with their offspring and have little or no exposure to any source of external stimulation. As a consequence, be the information transfer mechanism behavioral [11] and/or hormonal [5], dams are likely to reflect a rather non-challenging environment to their progeny. Conversely, their offspring are generally exposed to multiple challenges in adulthood (e.g. single housing, regrouping, injections, food deprivations, experimental testing, etc.) to which they are likely not prepared to respond [6]. Each of these events may elicit an elevation of stress-related hormones, which may ultimately result in maladaptive responses due to an excessive hypothalamic-pituitary-adrenal- (HPA) axis activation [13]–[15]. Thus, an individual that is not ready to respond to multiple challenges, might develop a phenotype that is non-adapted to the constraints of the laboratory setting. Whereas a natural population can be hypothesized to show a normal distribution among a series of outcome measures, a population in which maladaptive phenotypes are over-represented can show an abnormal probability distribution [4]. Conversely, a population in which maladaptive phenotypes are absent or negligible should exhibit a more “normal” probability distribution than a standard “control” population.

Robust experimental evidence demonstrates that a stimulating neonatal environment may reduce the stress and fear responses associated with adult testing in rodents. In particular, converging evidence shows that compared to control conditions moderately challenging neonatal environments, in the form of brief periods of maternal separation [16], increased foraging demands [17] or maternal corticosterone (CORT) supplementation via drinking water [18], reduce later stress and fear responses in adult rats. In particular, moderate levels of corticosterone supplementation have been proposed to constitute “a mild psychic stress” [18] to both dams and pups. Leonhardt and colleagues [19] recently showed that modest environmental perturbations in the form of ‘purely psychological stressors’ (1-hr strobe light exposure, forced foraging and wet bedding) induce peak maternal corticosterone levels noticeably similar to those observed in dams supplemented with corticosterone in the drinking water [18]. At a molecular level, these effects are most likely mediated via an increase in glucocorticoid receptor gene expression [11], [20], [21] and/or an increase in GABA-A and central benzodiazepine receptor levels [22]. We hypothesize that the reduced stress and fear responses associated with neonatal challenges might result in a lower random activation to experimental testing. This effect would ultimately increase the ‘signal-to-noise ratio’, increase the reproducibility of experimental findings and, finally, reduce the number of subjects needed to detect significant effects in behavioral and endocrine studies.

However, this form of phenotypic induction seems to follow a U-shaped curve whereby stronger neonatal challenges, in the form of longer mother-offspring separations and highly demanding foraging conditions, induce a phenotype that resembles the control profile in behavioral fearfulness and HPA activation [5], [16], [17], [23]. Finally, elevated dosages of neonatal corticosterone through maternal drinking water seem to have detrimental effects on immune competence in the mouse offspring [24].

Here we tested the hypothesis that, compared to control conditions (standard animal facility rearing, AFR) and to high levels of maternal corticosterone (H-CORT) administration (mimicking stronger neonatal stimulation), low levels of maternal corticosterone (L-CORT) would reduce within-group variation in a standardized test battery encompassing the behavioral, immune, and endocrine domain. Corticosterone was supplemented to maternal drinking water during the first week of lactation. The test battery was meant to assess, in independent groups of adult naïve subjects, general locomotion, pain perception, anxiety-like behavior during free-choice exploration of a novel environment, corticosterone response to a 25-min restraint stress and response to *Brucella* infection.

It has been demonstrated that neonatal manipulations often modify absolute levels of maternal care [11], [23]; environment dependent variations in maternal care have been in turn proposed to be the true mediator of the observed adult offspring stress and fear responses [11]. In order to address whether corticosterone administration would modify mother offspring interaction, we carefully scored maternal behavior through a detailed ethogram [25].

## Results

### Fluid Intake

To control for the effects of corticosterone on fluid intake, we measured the amount of solution drunk throughout treatment. Whereas, as expected, fluid intake rose throughout lactation (day, F_6,126_ = 27.5, P<0.01), this rise was indistinguishable between dams of the three groups (treatment, F_2,21_ = 0.6, NS, data not shown).

### Maternal care

**Figure 1:**
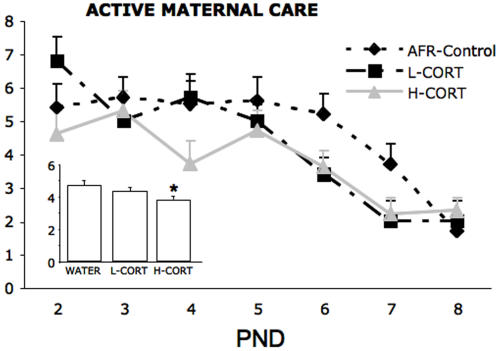
Effects of maternal CORT on maternal behavior. Daily frequency (mean/hour±SEM) of active maternal care shown by control, CORT-L and CORT-H dams (N = 8 dams per group); daily scores are based on 3 daily one-hour sampling sessions (10 observations/hour). Inset: total levels of active maternal care across days (mean frequency/hour±SEM). Scores from each of 3 daily one-hour sampling sessions were averaged across postnatal days 2 to 8.

Active maternal care: maternal behavior was observed throughout the treatment period (fig. 1). Overall levels of active maternal care steadily declined in the three groups between PND 2-8, albeit at a different rate (day, F_6,114_ = 13.9, P<0.01). Thus, dams drinking high levels of corticosterone showed reduced levels of active maternal care compared to AFR dams (treatment, F_2,19_ = 3.8, P<0.05, P<0.02 in post hoc test). Dams drinking low levels of corticosterone showed intermediate levels of active maternal care compared to AFR and H-CORT dams, though differences were not statistically significant (fig. 1 inset P = 0.20 in post-hoc tests).

Prone Nursing: absolute levels of prone nursing mirrored levels of active maternal care. Thus, prone nursing steadily increased across days (day, F_6,114_ = 16.9, P<0.01). Furthermore, although the difference was not significant, H-CORT dams showed slightly higher levels of prone nursing compared to AFR subject, whereas L-CORT dams showed intermediate levels (treatment, F_2,19_ = 2.4, P = 0.10).

Contact with pups: total time spent in contact with pups generally declined between PND 2-8. Although the main effect of day was not significant (day, F_6,114_ = 2.8, P<0.08), levels of contact with pups were higher on day 1 compared to day 8. However, total time spent with pups did not differ between the three groups (treatment, F_2,19_ = 0.3, NS), thus suggesting that the reduction in maternal behavior was specific to levels of active maternal care.

Self-directed activities: activity out of the nest mirrored the time spent in contact with pups. Thus, time spent in self-directed activities increased between PND 2-8 (days, F_6,114_ = 2.1, P = 0.05). However, dams from the three groups showed indistinguishable levels of self-directed activities (treatment, F_2,19_ = 0.5, NS).

Locomotion out of the nest: dams from the three groups showed indistinguishable levels of locomotion out of the nest (treatment, F_2,19_ = 0.8, NS).

### Behavioral, endocrine and immune responses in adult offspring

#### Inter-individual variation

**Figure 2:**
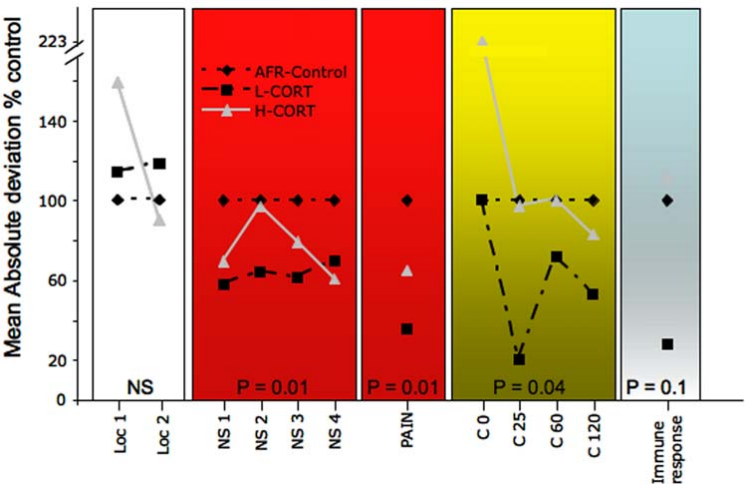
Effects of maternal CORT on inter-individual variation (percentage ratio). Within-group variation in general locomotion, response to novelty, pain response, corticosterone levels (in basal condition and 25, 60, 120-min following the beginning of a 25-min restraint stress) and immune response shown by adult AFR, L-CORT and H-CORT offspring. Variability has been calculated as the MAD (mean absolute deviation from the group average per each group) and here is expressed as the percentage ratio, whereby control subjects contribute the reference value (real values are reported in fig. 3). Each data point represents the variation in the different tests: Loc 1, locomotion minutes 1–10; Loc 2, locomotion minutes 11–20; NS1-2-3-4, Novelty seeking time bins 1–5, 6–10, 11–15, 16–20, respectively; Pain, paw-lick latency; C0, 25, 60, 120, corticosterone levels at time 0, 25, 60, 120; Immune Response, *Brucella* colony forming units (CFU). Significance levels are reported for the comparison L-CORT vs. control groups (N = 6-8 per group per test). Since main aim of the study was to demonstrate that moderate neonatal stress would result in adult subjects characterized by reduced inter-individual variation, significance values are reported only for the AFR controls vs. L-CORT comparison. See text for further details.

**Figure 3:**
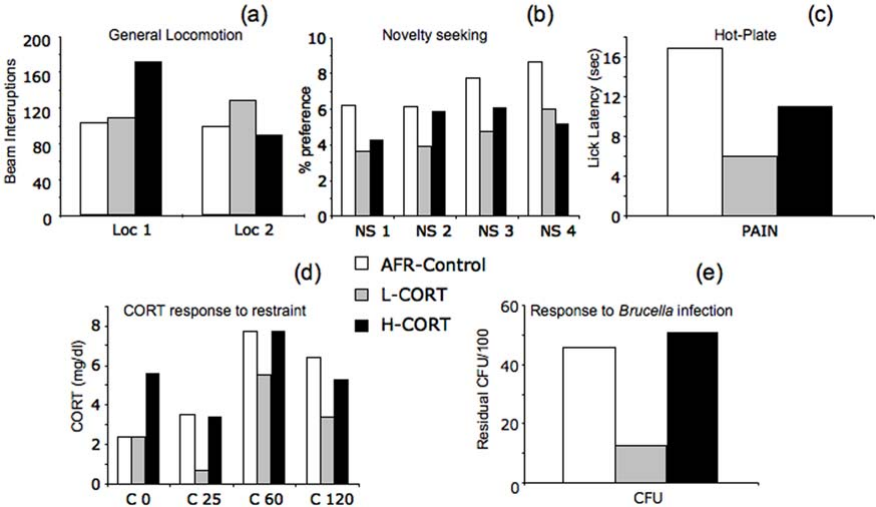
Effects of maternal CORT on inter-individual variation (real values). Within-group variation calculated as the MAD (mean absolute deviation from the group average per each group) in general locomotion (a), novelty seeking (b), pain response (c), corticosterone levels (in basal condition and 25, 60, 120-min following the beginning of a 25-min restraint stress, d) and immune response (e) shown by AFR controls, L-CORT and H-CORT adult offspring. Each bar represents the variation in the different tests: Loc 1, locomotion minutes 1–10; Loc 2, locomotion minutes 11–20; NS1-2-3-4, Novelty seeking time bins 1–5, 6–10, 11–15, 16–20, respectively; Pain, paw-lick latency; C0, 25, 60, 120, corticosterone levels at time 0, 25, 60, 120; Immune Response, *Brucella* colony forming units (CFU) (N = 6-8 per group per test).

The main aim of the present study was to demonstrate that compared to standard control conditions moderate neonatal stimulation might substantially reduce adult inter-individual variation in a number of parameters. To this aim, we performed a test of homogeneity of variances (Levene test) on all the parameters considered. In figure 2 we report the within-group variation observed in all the parameters analyzed. Variability is expressed as a percentage ratio, with the AFR control within-group variation as the reference value (actual values are reported in fig. 3). Within-group variation was indistinguishable among the three groups of mice for levels of general locomotion (treatment, F_2,39_ = 0.9, NS). Conversely, subjects of the three groups showed differential variability in novelty preference (treatment, F_2,39_ = 3.4, P<0.05), corticosterone response to restraint (treatment, F_2,16_ = 5.7, P<0.02), pain perception (treatment, F_2,19_ = 3.8, P<0.04) and response to bacteria infection, though the latter main effect just failed to reach statistical significance (treatment, F_2,15_ = 2.5, P = 0.1) (fig. 2).

In agreement with our predictions, apart from general locomotion, compared to AFR controls, L-CORT subjects showed a significantly reduced variation in all the parameters considered (figs. 2–3). Noticeably, whereas corticosterone basal levels showed similar variation between AFR control and L-CORT subjects, hormone levels following restraint stress were more variable in control subjects than in L-CORT mice. This finding supports the view that the elevated inter-individual variation observed in control mice in a number of tests may stem from an abnormal reaction to experimental challenges. Compared to AFR controls, H-CORT subjects showed indistinguishable inter-individual variability in general locomotion, pain perception and corticosterone and immune response. Conversely, compared to L-CORT subjects, H-CORT mice showed increased variation in corticosterone response (p<0.01) and a tendency towards increased variation in novelty preference, pain perception and response to pathogens (p = 0.10).

#### Frequency distribution

**Figure 4:**
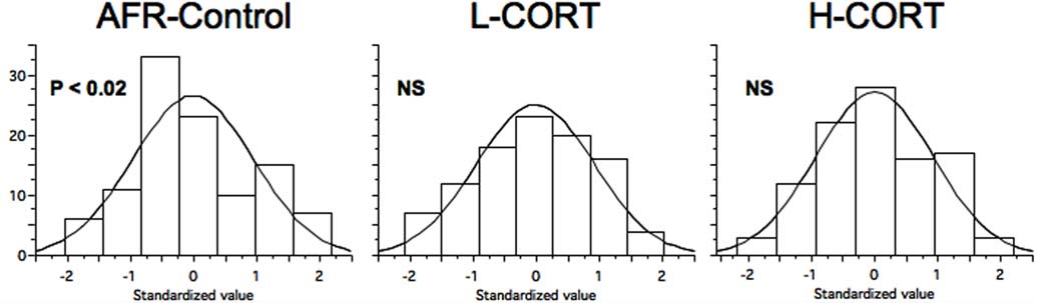
Effects of maternal CORT on frequency distribution. Frequency distribution of data from the novelty preference, hot plate, corticosterone response to restraint and immune response. Since these parameters showed a differential within-group variation, we performed an analysis to test whether reduced variation would also affect the data distribution. Data from all tests were standardized (see text), pooled and finally analyzed for normality distribution through Shapiro-Wilks test. Whereas data obtained in control subjects showed a significant deviation from normality, L-CORT and H-CORT subjects showed a normal distribution of data. Histograms represent the frequency distribution of data and the bell-shaped line represents the normal comparison.

It might be argued that reduced variation could beget a narrow distribution (e.g. characterized by abnormal kurtosis), non-representative of the normal population, which is the general target of behavioral neuroscience studies. In Fig. 4 we report the frequency distribution of all the data for which we observed a statistically significant difference in within-group variation. We transformed all the raw data into standardized Z-values. This transformation results in values characterized by a distribution with mean = 0 and SD = 1, yet it maintains the original shape of the data distribution. Following this transformation we controlled for the normality distribution assumption through the Shapiro-Wilks test. Fig. 4 thus offers a general view of the distribution of data. Whereas control subjects showed a small, yet significant deviation from normality (W = 0.959, P = 0.014), both L-CORT (W = 0.975, P = 0.3) and H-CORT (W = 0.980, P = 0.5) subjects showed a Gaussian distribution.

#### General locomotion

Levels of locomotion did not differ between the three groups neither in adolescence nor in adulthood (treatment, H = 2.0, df = 2, NS). However, adult mice showed a significant 12% increase in locomotor activity levels compared to adolescent subjects (age, U = 131.0, P<0.01).

#### Novelty seeking in adolescence and adulthood

**Figure 5:**
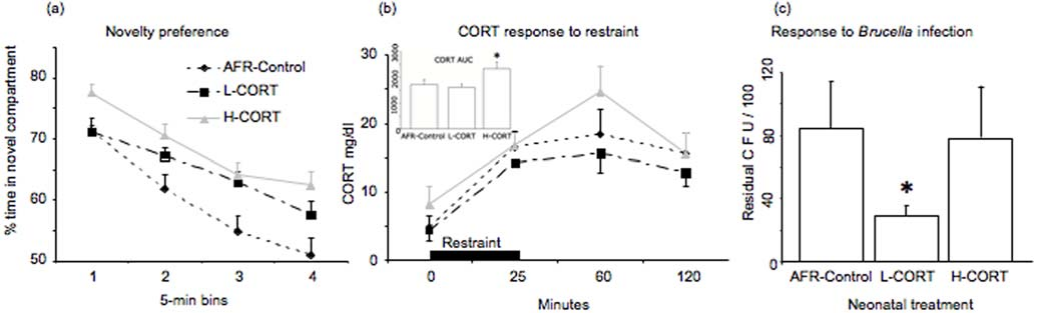
Effects of maternal CORT on behavioral, endocrine and immune responses in adult offspring. (a) Novelty preference: Mean (+S.E.M.) percentage of time spent in the novel compartment. After 5 min spent in the familiar compartment, a partition was removed, and mice were allowed free-choice access to a novel compartment of the apparatus for a single 20-min session (N = 16 mice per group); (b) Corticosterone levels in basal condition and 25, 60, 120-min following the beginning of a 25-min restraint stress. Inset: corticosterone area under the curve (expressed in mg×min/dl) * p<0.05 compared to H-CORT subjects; (c) Immune response: Colony forming units in the spleen two weeks after a standardised virulent challenge. Data analysis has been performed on Log-transformed values. For the sake of reading, real values are reported in the figure. * p<0.05 compared to AFR controls.

Following partition opening, all mice showed a marked preference for the novel environment compared to the familiar one. Although the novel compartment was generally preferred over the familiar one, the percent preference gradually declined throughout the test session (fig. 5a). As expected, adolescent mice showed increased preference for the novel compartment compared to adults (age, χ^2^ = 4.95, df = 1, P<0.03). Maternal CORT increased the preference for the novel environment both in adolescent and adult mice compared to AFR control subjects (treatment, χ^2^ = 9.48, df = 2, P<0.01, see fig 5a). The effects of CORT were independent of age (age×treatment χ^2^ = 0.64, df = 2, P>0.70). Thus, both adolescent and adult L-CORT and H-CORT mice showed increased preference for the novel compartment compared to AFR controls (P<0.05 in post-hoc tests). Additionally, H-CORT mice showed a tendency towards increased preference for the novel compartment compared to L-CORT subjects (P = 0.13).

#### Hot plate response

Adult AFR, L-CORT and H-CORT mice showed similar average paw-lick latency in the hot plate (36.1±7.7; 36.2±2.6; 40.5±5.0s respectively, treatment, H = 0.9, df = 2, NS).

#### Corticosterone response to restraint

Since non-parametric tests did not allow an analysis on observations taken at different time points, statistics were performed on the area under the curve, calculated using the trapezoidal rule. Mice from the three groups showed a differential corticosterone response (treatment, H = 6.2, df = 2, P<0.04, fig 5b inset). In particular, adult H-CORT mice showed increased area under the curve response compared to both L-CORT (U = 5.0, P<0.05) and AFR subjects (U = 8.0, P<0.04). Finally, AFR subjects were indistinguishable from L-CORT mice (U = 15, NS). Data inspection suggested that, whereas mice from the three groups showed similar circulating levels of CORT under basal conditions, immediately after restraint and 120-min following the beginning of restraint, H-CORT mice showed higher CORT response 60-min after the beginning of restraint (see Fig. 5b).

#### Immune response to *Brucella*

Bacterial clearance of adult AFR, L-CORT and H-CORT mice is shown in Fig. 5c. A significantly lower level of spleen colonization was observed two weeks post-infection in L-CORT mice compared to AFR controls (F_1,8_ = 6.5, P<0.04). Similarly, H-CORT mice seemed to show a higher level of spleen colonization compared to L-CORT subjects, yet this difference failed to reach statistical significance (F_1,8_ = 3.8, P = 0.08). We suggested that an adequate level of neonatal stimulation would ‘adjust’ newborn offspring to respond to adult challenges. This effect would then also modify immune reactivity due to the modulating effects of corticosteroids on the immune response. An enduring elevation of the HPA axis may, in the long term, persistently reduce immune adaptive capacities [13], [15]. The observed immune competence shown by L-CORT subjects matches these predictions.

## Discussion

Adult male mice reared by dams supplemented with low doses of corticosterone showed reduced inter-individual variation in behavioral, endocrine and immune parameters compared to both AFR controls and adult mice reared by dams exposed to high doses of corticosterone. Additionally, in agreement with our predictions, compared to AFR controls, adult L-CORT mice showed increased preference for a novel environment and improved resistance to bacteria infection. We hypothesized that mice exposed to mild neonatal stress would show reduced activation to the procedures associated with both general husbandry and experimental testing and in turn result in an experimental group characterized by reduced inter-individual variation. The observed results are in agreement with our hypothesis. Only in general locomotion was within-group variation unaffected by neonatal treatments: this finding further supports the view that locomotor activity represents a robust parameter, virtually unaffected by environmental variation [26]. Recently Wahlsten and colleagues demonstrated that strain differences in general locomotion ‘are highly replicable across laboratories and even decades’ [26].

The reduction in behavioral fearfulness shown in the novelty preference test by L-CORT mice supports the view that these subjects are less perturbed when faced with a novel situation. In contrast with our expectations, L-CORT and control subjects showed indistinguishable corticosterone response to restraint. Several factors may account for this effect. We cannot exclude the possibility that either we failed to observe the expected phenotype due to an absence of effects or that compensatory mechanisms other than glucocorticoid receptor expression (e.g. hypothalamic CRF levels, adrenal sensitivity to ACTH etc.) may have leveled-out the expected differences. Finally, since restraint is able to elicit a near-maximal corticosterone elevation, it is plausible that ceiling effects may have reduced the possibility to detect significant differences. Independent of the specific mediating mechanisms, the elevated inter-individual variation observed in control subjects in response to restraint may partly explain the absence of differences between L-CORT and control subjects. This is yet another indication that a disproportionate signal-to-noise ratio might hamper the detectability of effects. It also further suggests that designing novel housing and breeding procedures aimed at reducing inter-individual variation might increase the validity of experimental models and substantially decrease the number of subjects needed for animal experiments.

Furthermore, since these findings were observed in neuro-endocrine response and in behavioral performance as well, this study suggests that, rather than being limited to the behavioral realm, abnormal within-group variation can be the norm among a spectrum of variables and that these conclusions may well apply to a number of domains.

Of extreme relevance in this study is the fact that, upon infection with *B. melitensis*, L-CORT mice showed reduced susceptibility–suggesting that treatment is able to induce modifications in the immune response toward bacterial infections– and reduced within-group variation in spleen colonization. Increased protection suggests that L-CORT mice had greater ability to restrict infection compared to AFR control subjects, thus fostering the view that neonatal stimulation might benefit laboratory animal welfare. It remains to be further investigated whether the reduction in inter-individual variation in immune response observed in L-CORT subjects is dependent on a ‘floor’ effect due to the observed significant reduction in absolute values. Although we cannot rule out this possibility, we believe that the reduction in within-group variation is unlikely to reflect an artifact. In fact, we observed dissociation between absolute values and inter-individual variability in many of the other parameters considered. For example, corticosterone response to restraint and pain perception were identical in absolute values but different in variation; conversely, although H-CORT mice showed a differential corticosterone response compared to both the other groups, their variation was identical to AFR but different from L-CORT. Preference for the novel environment was even more informative since L-CORT subjects showed intermediate response in absolute values compared to AFR and H-CORT, yet they showed reduced variation compared to both the other groups.

H-CORT subjects constitute a relevant group to show that the effects of neonatal corticosterone, rather than being due to a non-specific action of corticosteroids supplementation, were specific to the dose selected. Just as a less variable HPA activation in response to conventional laboratory procedures mediates the reduced within-group variation, so also, from an evolutionary adaptive perspective, laboratory rodents should be adjusted to their specific adult conditions rather than to *some sort* of environmental challenge. In particular, current neonatal husbandry practices induce adult phenotypes that do not match the requirements of the adult environment (phenotypic mismatch [7]). If this hypothesis were true, there should be an optimal level of neonatal stimulation, which would induce an adaptive adult phenotype. Both higher and lower levels of stimulation should be reflected in maladaptive phenotypes. In agreement with these predictions, both AFR controls and H-CORT mice showed a differential inter-individual variation compared to L-CORT subjects. In the framework of adaptive plasticity, this finding has a further implication whereby present standards of animal facility rearing might foster appropriate animal models if adult subjects were tested in less challenging experimental conditions. For example, recent advances in automated behavioral phenotyping [27], [28] prefigure the possibility to study functional and dysfunctional physiological and behavioral development in the home cage, thus minimizing test-dependent arousal. Under these circumstances AFR bred subjects may well represent a better animal model compared to early challenged subjects, or at least beget similar results. In agreement with this hypothesis, in their original study, Crabbe and colleagues [1] observed that, in the home cage ethanol-drinking test, the main effects of laboratory-site and its interaction with strain were negligible. Unlike the other behavioral tests, ethanol preference test ‘extended over 6 days in the home cage and involved a bare minimum of handling mice by the experimenter’ [1]. This finding supports the view that AFR subjects might represent a valid animal model under conditions of minimal experimental disturbance.

It is important to reiterate that our aim was not to abolish inter-individual variation. Our original hypothesis was that ‘control’ subjects exhibit an abnormal variation and that neonatal stimulation might rectify this abnormality. The observation that experimental data obtained in L-CORT subjects meet the normality distribution assumption further supports this view. We conclude that a pharmacological stimulation of the HPA axis, mimicking a neonatal environmental challenge resulted in a ‘normal’ distribution of data thus guaranteeing the presence of individual variation. At the same time the average 44% reduction in within-group variation observed in this study discloses unprecedented avenues towards the improvement of our current animal models and the reduction of the number of subjects employed in animal experimentation. For example, the number of subjects (n) needed to detect a significant effect in a student T-test is equal to n = 2(kσ)^2^, where σ is within-group variation, and K is a function of type 1 and type 2 error probabilities (α and β), and of the difference between means (Δ). The observed 44% reduction in σ would reduce the number required to achieve the same statistical power by approximately 65% (i.e. 35 subjects instead of 100).

The possibility that the adaptive mechanisms and the logic described in this study may apply to captive mammals housed in different facilities (e.g. zoos and farms) awaits further testing.

Albeit expensive and time consuming, future studies aimed at re-defining neonatal husbandry strategies may substantially improve immune resistance, benefit the quality of experimental data and decrease the number of subjects used in experimental trials.

## Materials and Methods

### Subjects

Pregnant outbred CD-1 female mice (purchased from Harlan, 20050 Correzzana, MI, Italy) were housed in standard polycarbonate cages (33.0×13.0×14.0 cm) with sawdust bedding and *ad libitum* water and rodent pellets (Enriched standard diet purchased from Mucedola, Settimo Milanese, Italy). They were maintained on a reversed 12:12 h light:dark cycle (lights on at 1900 h) with temperature at 21±1°C and relative humidity of 60±10%. Dams were inspected daily at 0930 h for delivery and day of birth was designated as postnatal day 1 (PND 1). Between delivery and weaning all subjects were kept under standard facility rearing conditions (cage cleaning once a week). Litters were not culled until weaning and dams that delivered less than six pups or litters in which male to female ratio was heavily skewed in one or the other direction (more than 75% of same sex pups) were excluded from the experiment. Due to a failure to meet these requirements 6 dams (two dams per group) were excluded from the experiment (n = 8 dams per group). Only male mice were used in the study. To avoid litter effects, no more than one mouse per dam was used for each experimental procedure, resulting in 6-8 mice per group per test (see table 1 for details about littermates allocation to experimental testing). At PND 21, offspring were weaned and groups of three male subjects were formed. Post-weaning cage composition was counterbalanced across groups: thus, each cage consisted of one male mouse per neonatal treatment group (i.e. one AFR, one L-CORT and one H-CORT subject). Mice were marked at weaning using permanent marker.

**Table 1:** Allocation of littermates to experimental tests

Test	Mouse 1	Mouse 2	Mouse 3	Mouse 4	
Locomotor activity+Novelty (adolescents, PND 37-41)		X			
Locomotor activity+Novelty (adults, PND 77-81)			X		
Hot-plate (PND 65)				X	
Corticosterone response (PND 115)	X				
Immune resistance (PND 90-104)		X			

Each dam provided four male mice to the study. Littermates were tested according to the schedule reported in the table. E.g. mouse 2 performed the novelty-seeking test between PND 37-41 and was then assessed for immune response between PND 90-104. This schedule was adopted in order to maximize the information obtained by each mouse and to minimize the potential carry-over effects due to previous testing. In one case we could not use naïve mice; therefore we first exposed mice to the least stressful procedure (novelty seeking) and then waited a long time before the second test (immune resistance).

All animal handling and experimental procedures were performed according to European Communities guidelines (EC Council Directive 86/609), Italian legislation on animal experimentation (Decreto L.vo 116/92) and NIH guide for the care and use of laboratory animals.

### Corticosterone administration

The day of birth was counted as PND 1, and, starting on PND 2, 10 mothers were maintained on tap water (AFR control), 10 mothers had free access to a solution of 33 µg/ml of corticosterone 21-sulfate (Sigma, St Louis, Mo., USA, L-CORT) and 10 mothers had free access to a solution of 100 µg/ml of CORT (H-CORT). Other than solution in the drinking bottle (water or CORT) environmental conditions were identical among groups. The treatment lasted until PND 8. The treatment period was selected based on the following reasons. Classical neonatal manipulations (e.g. brief and long maternal separations) have been shown to modify offspring phenotype when applied during the first week of life, and longer periods are as effective [11]. Mouse HPA reactivity during the first week of life is constantly down regulated [29]. The doses were based on previous literature in rats [18] and mice [24]. Compared to rats, mouse dams show a four-fold higher water intake (see [18] and [30] for a comparison). We therefore selected the low dose in order to have comparable levels of corticosterone intake per g of body weight in dams between our study and the previous studies performed by Catalani and colleagues [20]. The doses selected resulted in a daily average corticosterone intake of 2,7±0,1 mg/mouse and of 0,9±0,03 mg/mouse in the H-CORT and in the L-CORT group, respectively. This administration route in mice results in a robust increase in corticosterone levels both in the dams and in the pups [24]. Importantly, corticosterone raise can be observed both in the serum and in the milk of the lactating female. As shown by a comparison between the studies performed by Catalani and colleagues [18] and Leonhardt and colleagues [19], this procedure produces plasma levels of the hormone in the range of those produced by a mild psychic stress (from 4.3+/−0.5 to 9.5+/−1.8 micrograms/100 ml in the dams, and from 0.7+/−0.1 to 1.2+/−0.2 micrograms/100 ml in the pups at 10 days of lactation).

### Maternal behavior

The behavior of the dams was scored between PND 2-8 according to a detailed ethogram [25]. Maternal care was observed daily for three 1-h sessions, distributed during the dark phase (starting at 900, 1200 and 1500 hours respectively), by instantaneous sampling at an interval of 6 min (30 samples per day for each dam). In the present paper, the following behaviors are reported:

#### Active maternal care

high kyphosis, low kyphosis, partial kyphosis, and licking (but not prone nursing and supine nursing; cf. [23]).

#### Prone nursing

The dam lies flat on top of the pups with little or no limb support.

#### Contact with pups

time spent by the dams in contact with pups.

#### Self-maintenance

the dam is not in any form of physical contact with her pups. This behavioral category comprised feeding and drinking, active behavior outside the nest, resting and self-grooming (licking, scratching and washing of the head and body).

#### General locomotion out of the nest

dams wondering about the cage and not in any form of physical contact with pups.

### Response to novelty

To evaluate mice response to a novel environment in a test that has been validated in our lab and for which we developed a standardized protocol in mice [31], both adolescent (PND 37-41) and adult (PND 77-81) subjects were first familiarized for three days with a compartment of an apparatus and then allowed to freely explore both the familiar and the unfamiliar compartment (see SI). Novelty preference is considered an inverse index of anxiety whereby an anxious mouse tends to avoid a novel and unknown environment [32], [33].

Novelty seeking was evaluated in both adolescent (pnd 37-41) and adult (pnd 77-81) subjects. Adolescent subjects have been proposed to be characterized by higher variation compared to adults [34], [35]. Furthermore, one of the authors (G.L.) has a long-held experience in adolescent mouse behavior and has previously observed [31] spontaneously elevated levels of novelty in adolescent mice compared to adults. Therefore, analyzing novelty in adolescent and adult subjects would serve two objectives: (i) test whether adolescent and adult subjects do indeed show differential variation and how this difference can be modified by neonatal corticosterone treatment; (ii) evaluate whether novelty preference test is valid and resistant to the challenge of repeated testing approximately 10 years after the original observation.

### Apparatus

The experimental apparatus consisted of an opaque Plexiglas rectangular box with smooth walls, which was subdivided into two compartments (20×14×27 cm). The door between the two compartments could be closed by means of a temporary partition. One distinctive visual cue was associated with each compartment. One compartment had white walls and black floor, whereas the other one had black walls and white floor. Each compartment was provided with four pairs of infrared photobeams, placed on the wall at few cm from the floor, 5.5 cm apart. Each beam interruption eventually caused by mice was recorded by an IBM computer equipped with dedicated software. The following measures were obtained automatically: 1) time spent in each compartment, 2) activity rate in each compartment (number of beam interruptions/second), 3) frequency of passages between the two compartments (number of passages/minute), and 4) latency (time between the opening of the partition and the first entrance in the novel compartment). The whole session was automatically subdivided into 5-min intervals.

### Procedure

One compartment of the apparatus (black floor) was the familiar one, whereas the other compartment was the novel one [31]. The preference for the novel compartment has been shown to be independent of the environmental cues provided [36].

Day 1,2,3: Familiarization. Animals were placed for 20 min in the familiar compartment of the apparatus for a 20-min session.

Day 4 (wash-out). A wash-out interval of 48 h was introduced between the last day of familiarization and the test.

Day 5: Novelty preference test. Animals were weighed and immediately placed in the familiar compartment, for a 5-min session. The partition separating the two compartments of the apparatus was then removed, and mice were thus allowed to freely explore the whole apparatus (both the familiar and the novel sides) for 20 min.

### Hot plate

The apparatus consisted of a metal plate 25×25 cm (Socrel Mod. DS-37, Ugo Basile, Italy) heated to a constant temperature of 48±0.1°C, around which a plastic cylinder 20 cm in diameter, 18 cm high was placed. The temperature was chosen so as to minimize suffering and distress in the experimental subjects. The latency (s) was recorded from the moment the animal was inserted onto the plate up to when it first licked one of its paws. The measurement was terminated if the latency exceeded the cut-off time of 60s (e.g. [37]). Hind-paw liking has often been adopted as the main end-point. However, several other authors reported recorded ‘the latency to the first sign of discomfort’ [38]. Additionally, the specificity and sensitivity of the test has been reported to be increased by measuring the reaction time of the first evoked behavior regardless of whether it is paw-licking or jumping [39]. Furthermore, the key issue addressed in our study was the reproducibility of experimental findings. We therefore decided to adopt testing strategies in which the experimenter performing the test had a substantial degree of confidence thereby minimizing the possibility to introduce experimenter-dependent variability. We therefore chose a procedure that we have already adopted several times in the past (e.g. [37], [40]). Finally, this protocol also minimizes the time that mice spent on the hot plate and the suffering associated with it.

### Plasma corticosterone response to restraint stress

#### Plasma sampling

At the age of about 16 weeks, from each litter one male was taken from the home cage, carried by a familiar experimenter to an adjacent room and bled (t0) from the tail (0.2–0.4 ml collected into prechilled ethylenediamine tetraacetic acid (EDTA)-coated tubes; Microvette; Sarstedt, Sevelen, Switzerland) by tail incision [41] within 2 min from entering the colony room to obtain a blood sample for analysis of basal plasma corticosterone. Subsequently, mice were placed in a transparent Plexiglas restraint tube (2.8 cm in diameter) of adjustable length for 25 min to induce a stress response. After 25 min, they were bled from the same tail incision for a second time (t25) to obtain blood samples for analysis of the peak stress response before release from restraint and transport back to the home cage. At times t60 and t120, mice were again transported to the adjacent room, bled from additional tail incision for a third and fourth time to obtain blood samples for analysis of recovery from the stressor, and returned to the colony room. Blood was sampled between 1030 and 1300 hours. Samples were cool centrifuged, and the plasma stored at −80°C until assayed. CORT was measured by a commercial radioimmunoassay (RIA) kit (ICN Biomedicals, Costa Mesa, CA). Sensitivity of the assay was 0.125 mg/dl, inter- and intra-assay variation was less than 10 and 5%, respectively.

### Response to *Brucella*

In order to assess the effect of treatment upon the capability of mice to respond to an infectious disease we used an experimental model of infection due to *Brucella melitensis* [42]. The capability of mice from the three groups to restrict the spleen infection after a standardized virulent challenge was evaluated. Six mice per group were challenged i.p. with *B melitensis* 16 M, at 2×105 CFU each, at 40 dpv. Two weeks later, mice were killed by cervical dislocation and spleens were removed for bacteriological examination as described below. A mean value for each spleen count was obtained after logarithmic conversion.

#### Bacteriological examination

To detect *Brucella* organisms, spleens were aseptically removed from sacrificed mice, individually weighed, and diluted 1/10 (wt/wt) in sterile phosphate-buffered saline. Further dilutions were made, and 0.1 ml of each dilution was plated in triplicate onto BAS medium and incubated at 37°C for 5 days. The *Brucella* isolates were identified by Gram staining, colony morphology, and *Brucella*-specific PCR procedures.

### Statistical Analysis

Within-group variability was analyzed through Levene test for homogeneity of variances. The general model for Levene test depended on the parameter of interest. For analysis of novelty seeking, the model was 2 age×3 treatments×4 time bins. For analysis of plasma levels of corticosterone, it included 3 treatments×4 time points. Finally, for analysis of hot plate and immune response, treatment (3 levels) was the only factor analyzed. Age and treatment were between-litter factors while time bins and time points were within-litter factors. To evaluate the frequency distribution, data from all tests were standardized, pooled and finally analyzed for normality distribution through Shapiro-Wilks test.

Most of our data failed to meet the homoschedasticity assumption. Therefore, data analysis on absolute values was based on non-parametric methods, which do not allow the analysis of complex split-plot designs. Repeated measures were pooled using a unitary parameter, the average of the four time-bins for the response to novelty test and the area under the curve for corticosterone response. We then performed Kruskal-Wallis analysis of variance (ANOVA) followed by Mann-Whitney U test for paired comparisons. For response to novelty, the Kruskal-Wallis ANOVA was performed on the six treatment×age groups, followed by orthogonal chi-square partitioning to test main effects of treatment and age, and their interaction. Immune parameters were log-transformed and analyzed through parametric ANOVA. Maternal care was analyzed by repeated-measures ANOVA for split-plot designs. The general model was 3 treatments×7 days×3 hours. Treatment was a between-litter factor while all other variables were within-litter factors.

## References

[1] Crabbe JC, Wahlsten D, Dudek BC (1999). Genetics of mouse behavior: interactions with laboratory environment.. Science.

[2] Wolfer DP, Litvin O, Morf S, Nitsch RM, Lipp HP (2004). Laboratory animal welfare: cage enrichment and mouse behaviour.. Nature.

[3] Tsai PP, Stelzer HD, Hedrich HJ, Hackbarth H (2003). Are the effects of different enrichment designs on the physiology and behaviour of DBA/2 mice consistent?. Lab Anim.

[4] Garner JP (2005). Stereotypies and other abnormal repetitive behaviors: potential impact on validity, reliability, and replicability of scientific outcomes.. Ilar J.

[5] Macri S, Wurbel H (2006). Developmental plasticity of HPA and fear responses in rats: a critical review of the maternal mediation hypothesis.. Horm Behav.

[6] Wurbel H (2001). Ideal homes? Housing effects on rodent brain and behaviour.. Trends Neurosci.

[7] Bateson P (2004). Developmental plasticity and human health.. Nature.

[8] Hager R, Johnstone RA (2006). Early experience and parent-of-origin-specific effects influence female reproductive success in mice.. Biol Lett.

[9] Calhoun JB (1962). Sci. Am..

[10] Smotherman WP, Bell RW, Bell RW, Smotherman Wp (1980). Maternal mediation of early experience.. Maternal Influences and Early Behavior.

[11] Meaney MJ (2001). Maternal care, gene expression, and the transmission of individual differences in stress reactivity across generations.. Annu Rev Neurosci.

[12] Pryce CR, Feldon J (2003). Long-term neurobehavioural impact of the postnatal environment in rats: manipulations, effects and mediating mechanisms.. Neurosci Biobehav Rev.

[13] Barnard CJ, Behnke JM, Sewell J (1996). Environmental enrichment, immunocompetence, and resistance to Babesia microti in male mice.. Physiol Behav.

[14] Nestler EJ, Barrot M, DiLeone RJ, Eisch AJ, Gold SJ (2002). Neurobiology of depression.. Neuron.

[15] Sapolsky RM (2004). Why zebras don't get ulcers..

[16] Plotsky PM, Meaney MJ (1993). Early, postnatal experience alters hypothalamic corticotropin-releasing factor (CRF) mRNA, median eminence CRF content and stress-induced release in adult rats.. Brain Res Mol Brain Res.

[17] Macrì S, Würbel H (2007). Environmental modulation of maternal behaviour and behavioural and HPA-responses in rats.. Anim Behav.

[18] Catalani A, Marinelli M, Scaccianoce S, Nicolai R, Muscolo LA (1993). Progeny of mothers drinking corticosterone during lactation has lower stress-induced corticosterone secretion and better cognitive performance.. Brain Res.

[19] Leonhardt M, Matthews SG, Meaney MJ, Walker CD (2007). Psychological stressors as a model of maternal adversity: Diurnal modulation of corticosterone responses and changes in maternal behavior.. Horm Behav.

[20] Catalani A, Casolini P, Scaccianoce S, Patacchioli FR, Spinozzi P (2000). Maternal corticosterone during lactation permanently affects brain corticosteroid receptors, stress response and behaviour in rat progeny.. Neuroscience.

[21] Cameron NM, Champagne FA, Parent C, Fish EW (2005). The programming of individual differences in defensive responses and reproductive strategies in the rat through variations in maternal care.. Neurosci Biobehav Rev.

[22] Caldji C, Francis D, Sharma S, Plotsky PM, Meaney MJ (2000). The effects of early rearing environment on the development of GABAA and central benzodiazepine receptor levels and novelty-induced fearfulness in the rat.. Neuropsychopharmacology.

[23] Macri S, Mason GJ, Wurbel H (2004). Dissociation in the effects of neonatal maternal separations on maternal care and the offspring's HPA and fear responses in rats.. Eur J Neurosci.

[24] Yorty JL, Schultz SA, Bonneau RH (2004). Postpartum maternal corticosterone decreases maternal and neonatal antibody levels and increases the susceptibility of newborn mice to herpes simplex virus-associated mortality.. J Neuroimmunol.

[25] Capone F, Bonsignore LT, Cirulli F, Maines M, Costa L, Reed D, Sassa S (2005). Methods in the analysis of maternal behavior in the Rodent.. Current Protocols in Toxicology.

[26] Wahlsten D, Bachmanov A, Finn DA, Crabbe JC (2006). Proc Natl Acad Sci USA.

[27] Galsworthy MJ, Amrein I, Kuptsov PA, Poletaeva II, Zinn P (2005). A comparison of wild-caught wood mice and bank voles in the Intellicage: assessing exploration, daily activity patterns and place learning paradigms.. Behav Brain Res.

[28] de Visser L, van den Bos R, Kuurman WW, Kas MJ, Spruijt BM (2006). Novel approach to the behavioural characterization of inbred mice: automated home cage observations.. Genes Brain Behav.

[29] Schmidt MV, Enthoven L, van der Mark M, Levine S, de Kloet ER (2003). The postnatal development of the hypothalamic-pituitary-adrenal axis in the mouse.. Int J Dev Neurosci..

[30] Biggerstaff S, Mann M (1992). Consummatory behaviors and weight regulation in pregnant, lactating, and pregnant-lactating mice.. Physiol Behav.

[31] Adriani W, Chiarotti F, Laviola G (1998). Elevated novelty seeking and peculiar d-amphetamine sensitization in periadolescent mice compared with adult mice.. Behav Neurosci.

[32] Griebel G, Belzung C, Misslin R, Vogel E (1993). The free-exploratory paradigm: an effective method for measuring neophobic behaviour in mice and testing potential neophobia-reducing drugs.. Behav Pharmacol.

[33] Roy V, Chapillon P (2004). Further evidences that risk assessment and object exploration behaviours are useful to evaluate emotional reactivity in rodents.. Behav Brain Res.

[34] Spear L (2000). The adolescent brain and age-related behavioral manifestations.. Neurosci Biobehav Rev.

[35] Laviola G, Macrì S, Morley-Fletcher S, Adriani W (2003). Risk-taking behavior in adolescent mice: psychobiological determinants and early epigenetic influence.. Neurosci Biobehav Rev.

[36] Bardo MT, Bowling SL, Robinet PM, Rowlett JK, Lacy M (1993). Role of dopamine D1 and D2 receptors in novelty-mantained place preference.. Exp Clin Psychopharmacol.

[37] Pieretti S, d'Amore A, Loizzo A (1991). Long-term changes induced by developmental handling on pain threshold: effects of morphine and naloxone.. Behav Neurosci.

[38] Plone MA, Emerich DF, Lindner MD (1996). Individual differences in the hotplate test and effects of habituation on sensitivity to morphine.. Pain.

[39] Carter RB (1991). Differentiating analgesic and non-analgesic drug activities on rat hot plate: effect of behavioral endpoint.. Pain.

[40] Pieretti Pieretti S, Dal Piaz V, Matucci R, Giovannoni MP, Galli A (1999). Antinociceptive activity of a 3(2H)-pyridazinone derivative in mice.. Life Sci.

[41] Fluttert M, Dalm S, Oitzl MS (2000). A refined method for sequential blood sampling by tail incision in rats.. Lab Anim.

[42] Pasquali P, Rosanna A, Pistoia C, Petrucci P, Ciuchini F (2003). Brucella abortus RB51 induces protection in mice orally infected with the virulent strain B. abortus 2308.. Infect Immun.

